# CRISPR/Cas9 mediated mutagenesis of *MORE AXILLARY GROWTH 1* in tomato confers resistance to root parasitic weed *Phelipanche aegyptiaca*

**DOI:** 10.1038/s41598-021-82897-8

**Published:** 2021-02-16

**Authors:** Vinay Kumar Bari, Jackline Abu Nassar, Radi Aly

**Affiliations:** 1 Department of Plant Pathology and Weed Research, Newe Ya’ar Research Center, Agricultural Research Organization (ARO), Ramat Yishay, Israel; 2 Present Address: Department of Biochemistry, School of Basic Sciences, Central University of Punjab, VPO-Ghudda, Bathinda, India

**Keywords:** Biotechnology, Molecular biology, Plant sciences

## Abstract

Root parasitic weeds infect numerous economically important crops, affecting total yield quantity and quality. A lack of an efficient control method limits our ability to manage newly developing and more virulent races of root parasitic weeds. To control the parasite induced damage in most host crops, an innovative biotechnological approach is urgently required. Strigolactones (SLs) are plant hormones derived from carotenoids via a pathway involving the *Carotenoid Cleavage Dioxygenase* (*CCD*) *7*, *CCD8* and *More Axillary Growth 1* (*MAX1*) genes. SLs act as branching inhibitory hormones and strictly required for the germination of root parasitic weeds. Here, we demonstrate that CRISPR/Cas9-mediated targted editing of SL biosynthetic gene *MAX1,* in tomato confers resistance against root parasitic weed *Phelipanche aegyptiaca*. We designed sgRNA to target the third exon of *MAX1* in tomato plants using the CRISPR/Cas9 system. The T_0_ plants were edited very efficiently at the *MAX1* target site without any non-specific off-target effects. Genotype analysis of T_1_ plants revealed that the introduced mutations were stably passed on to the next generation. Notably, *MAX1*-Cas9 heterozygous and homozygous T_1_ plants had similar morphological changes that include excessive growth of axillary bud, reduced plant height and adventitious root formation relative to wild type. Our results demonstrated that, *MAX1*-Cas9 mutant lines exhibit resistance against root parasitic weed *P. aegyptiaca* due to reduced SL (orobanchol) level. Moreover, the expression of carotenoid biosynthetic pathway gene *PDS1* and total carotenoid level was altered, as compared to wild type plants. Taking into consideration, the impact of root parasitic weeds on the agricultural economy and the obstacle to prevent and eradicate them, the current study provides new aspects into the development of an efficient control method that could be used to avoid germination of root parasitic weeds.

## Introduction

Parasitic plants are characterized as obligate or facultative parasites; they attach to either the shoot or the root, and they may be hemi or holoparasitic in nature^1^. Parasitic plants adopt different forms to invade host plants. Some, such as dodder (*Cuscuta* spp.) and mistletoe (*Arceuthobium* spp.) are aerial parts parasite, whereas Orobanchaceae such as *Orobanche* and *Phelipanche aegyptiaca* penetrate the underground root and represent one of the most destructive and great challenge for the agricultural economy^1,2^. *P.aegyptiaca* an obligate root parasitic plant causes great damage to economically important crops such as Solanaceae, Asteraceae, Fabaceae, Apiaceae and Brassicaceae plant families and affect total yields^3^. The life cycle of *P. aegyptiaca* have two stages, preparasitic and parasitic. The preparasitic stage involves seed preconditioning followed by germination of *P. aegyptiaca* seeds which is induced by presence of chemical inducer strigolactones (SLs) exuded by host plant roots^4^. However, the parasitic stage initiates with the parasite developing special projection or root like structure known as haustorium that directly penetrates the tissues of a host and absorbs nutrients and water^5^. After successful invasion and connection to the host root, the parasitic seedling grows into a bulbous structure known as tubercle from which a floral meristem emerges above the ground to produce flower and set seeds^6^.

SLs, a class of carotenoid-derived terpenoid lactones, is a plant hormone essentially required for inhibition of shoot branching and used as a signaling molecule for the rhizosphere microflora^7,8^. SL biosynthesis begins with *DWARF 27* (*D27*) which catalyzes the isomerization in *all-trans-β-*carotene, followed by the combined activity of *Carotenoid Cleavage Dioxygenases* (*CCDs*) 7 and *8*, leading to the production of carlactone (CL). First identified in *Arabidopsis, MORE AXILLARY GROWTH 1* (*MAX1*), encodes a cytochrome P450 monooxygenase *CYP711A* subfamily member that acts as a CL oxidase to convert CL into carlactonoic acid which is further converted into various SLs^9,10^. Previous studies reported that in rice a *MAX1* homolog catalyzes the conversion of CL to 4-deoxyorobanchol (4DO)^11,12^. A recent study, in tomato reported the direct conversion of carlactonoic acid into orobanchol by cytochrome P450, *SlCYP722C* which act as orobanchol synthase^13^. In the rhizosphere, SL acts as a host detection molecule for symbiotic arbuscular mycorrhizal fungi and induces the germination of root parasitic weeds^14,15^. The existence of various types of SLs is reported such as strigol, 5-deoxystrigol, sorgolactone, solanacol, dideoxyorobanchol, orobanchol and others, which can act as germination inducer for root parasitic weeds^16^. Host resistance to root parasitic plant *Striga* has been reported in crops with decreased SL production^17,18^.

The clustered regularly interspaced short palindromic repeats (CRISPR)-associated protein 9 and its derivative systems have become a powerful tool to induce various type of targeted mutagenesis at the genome level with high success in diverse organisms including tomato plants^19–21^. Cas9-mediated genome editing generates desired or random modifications at a specific target sequence depending on choice of Cas9 editor^22^. Cas9 nuclease induces site-specific double-strand DNA breaks at a targeted locus in the genome generating random modifications through non-homologous end-joining repair mechanisms, while Cas9 base editor has the capacity to replace the nucleotide at a specific target site^23,24^. The most frequently used CRISPR/Cas9 system, type II, has mainly three components: Cas9 nuclease, tracer RNA and crRNA. The 3′-end of the target sequence contains an NGG protospacer adjacent motif (PAM) that is recognized by the Cas9 protein^25^. CRISPR/Cas9 has been successfully demonstrated to edit the various plant genomes^26,27^. Here, in this study, we report the genome editing of *MAX1* in tomato plants using CRISPR/Cas9 provides resistance against root parasitic weed *P. aegyptiaca.*

## Results

### Targeted mutagenesis of *MAX1* gene in tomato using CRISPR/Cas9

**Figure 1:**
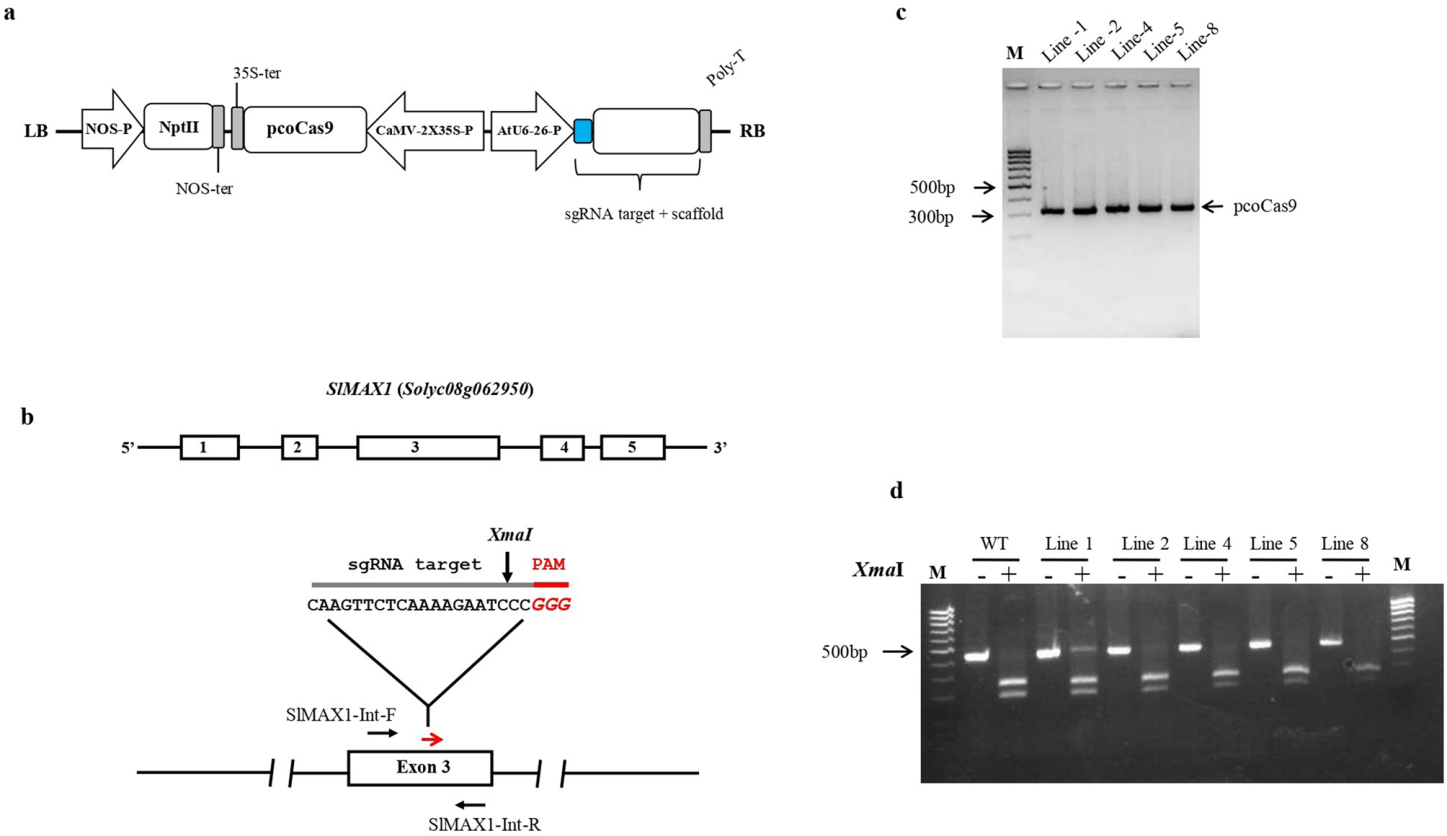
Schematic diagram of Cas9/sgRNA binary vector, target sites and targeted mutagenesis. (**a**) The strong constitutive CaMV-2 × 35S promoter was used to express plant codon-optimized Cas9 and the *Arabidopsis* U6-26 promoter was used to express MAX1-sgRNA. (**b**) Representation of the tomato *MAX1* genomic map and location of the sgRNA target site. The target sequence of sgRNA is shown in black color, the PAM is shown in red, black arrows indicate the primer (MAX1-Int-F and MAX1-Int-R) used for PCR amplification and red arrows indicate location of sgRNA target site, *Xma*I site marked with arrow. (**c**) Five independent T_0_ transgenic lines were generated by Agrobacterium-mediated transformation. The presence of the transgene was confirmed by using pcoCas9-specific primers (323 bp). (**d**) Detection of mutations at *MAX1* locus using PCR product restriction digestion. PCR fragments of MAX1-Cas9 targeted region in T_0_ lines from genomic DNA of transgenic plants were subjected to *Xma*I restriction digestion. Lines 1, 2, 4, 5 and 8 represent independent T_0_ transgenic plants; +, *Xma*I added; −, without *Xma*I. *Xma*I digested or non-digested PCR product were resolved using a 2% agarose gel for 60 min. Results shown that MAX1-Cas9 mutant line 1 contain equal amount of undigested and digested DNA, while other lines 2, 4, 5 and 8 show complete digestion, similar to control wild-type (WT). The full-length agarose gel blot represents the complete set of digested or non-digested PCR products run in independent wells of a single gel. Image was acquired using DNR Mini Lumi with UV light and Microsoft office was used to crop the image in appropriate size. M: DNA size ladder (100 bp).

**Figure 2:**
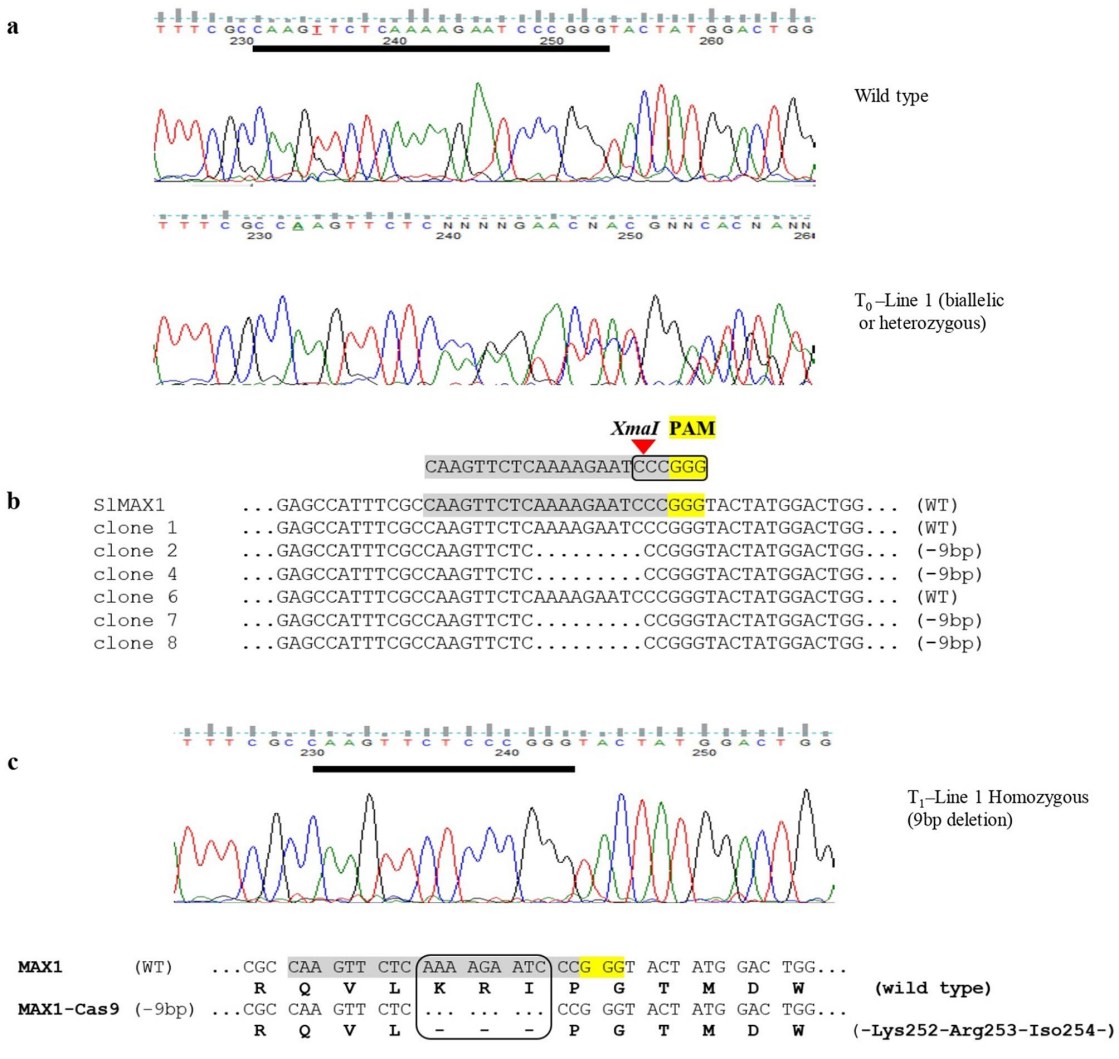
Identification of targeted editing events induced by CRISPR/Cas9 (**a**) DNA sequence chromatogram of MAX1-Cas9 targeted region, PCR product in the T_0_ lines shown multiple peaks. (**b**) PCR product sequence alignment of the MAX1-Cas9 T_0_ lines after TA cloning with the wild-type sequences (WT), deletion sizes (nt) are marked on the right side. (**c**) PCR product sequence alignment of the MAX1-Cas9 T_1_ homozygous lines which causes deletion of amino acid sequences (Lys252-Arg253-Iso254) from protein in mutated plant as compared to wild type.

To analyze the effectiveness of the genome editing approach and to generate host resistance against root parasitic weed *P. aegyptiaca,* we targeted the SL-biosynthesis gene *MAX1* (*Solyc08g062950*) in the host plant tomato using CRISPR/Cas9 system. The Cas9/sgRNA vector construct was designed to target the third exon of *MAX1* (position 778–797 bp in the coding region) having an *Xma*I restriction site located next to the protospacer adjacent motif (PAM) (Fig. 1a,b). Five transgenic lines 1, 2, 4, 5 and 8 were independently generated and presence of transgene was confirmed by kanamycin resistance and PCR amplification of pcoCas9 (Fig. 1c). To evaluate the types of mutation generated by Cas9/MAX1-sgRNA in T_0_ lines 1, 2, 4, 5 and 8, the flanking region was PCR amplified and the presence of indel mutations was tested by *XmaI* restriction digestion. Restriction analysis suggests that line 1 contained about equal amounts of undigested and digested DNA, in contrast other lines 2, 4, 5 and 8 seem non-mutants with most of the DNA digested similarly to wild type plants (Fig. 1d). DNA sequencing analysis of the target region from T_0_ line 1 shows multiple peaks possibly due to the presence of biallelic or heterozygous mutations (Fig. 2a), however in lines 2, 4, 5 and 8 no mutation was detected hence discarded. To explore the kind of mutation, existing in line 1, the *MAX1* target region was PCR amplified and directly cloned into a TA cloning vector and sequenced. Analysis of the DNA sequences revealed that T_0_ line 1 plant is heterozygous and contains in-frame deletion of 9 nt which causes removal of the amino acid (Lys-252, Arg-253 and Iso-254) in mutant as compared to wild-type protein after Cas9-mediated editing of the target (Fig. 2b,c).

Usually, T_0_ transgenic lines are somatic in nature, hence T_0_ line 1 was grown to maturity and self-pollinated to generate T_1_ plants. Genomic DNA was isolated from the T_1_ plants of line 1 and the target region of *MAX1* was again amplified using PCR primers flanking the target. When this analysis was performed on T_1_ plants of line 1, results were like those with the T_0_ line 1. At least 64 plants of the T_1_ lines were analyzed for genotype at the target site using sanger DNA sequencing. T_1_ plants from the T_0_ heterozygous line 1 were segregated according to mendelian law and the kind of mutation that existed was separated as 1:2:1 as reported previously (Table S1)^28^. The existence of T_0_ mutation by T_1_ plants suggested that the mutation resulting from CRISPR/Cas9 activity are highly stable in nature and inherited to the next generation without any alteration. Additionally, the presence of the transgene region (pcoCas9) was examined in the T_1_ generation plants. Our results demonstrated that 35.7% (5/14) T_1_ plants were detected to be transgene-free (Table S1). We have also analyzed potential off-target effects that occurred due to non-specific activity of Cas9, associated with MAX1-sgRNA in the tomato genome. At least two plants were selected from the T_1_ generations of MAX1-Cas9 edited homozygous lines. Sequencing analysis of PCR products from these regions revealed no change in the potential off-target sites (Fig. S1, Table S2).

### Alteration of morphological phenotype in MAX1-Cas9 mutated plants

**Figure 3:**
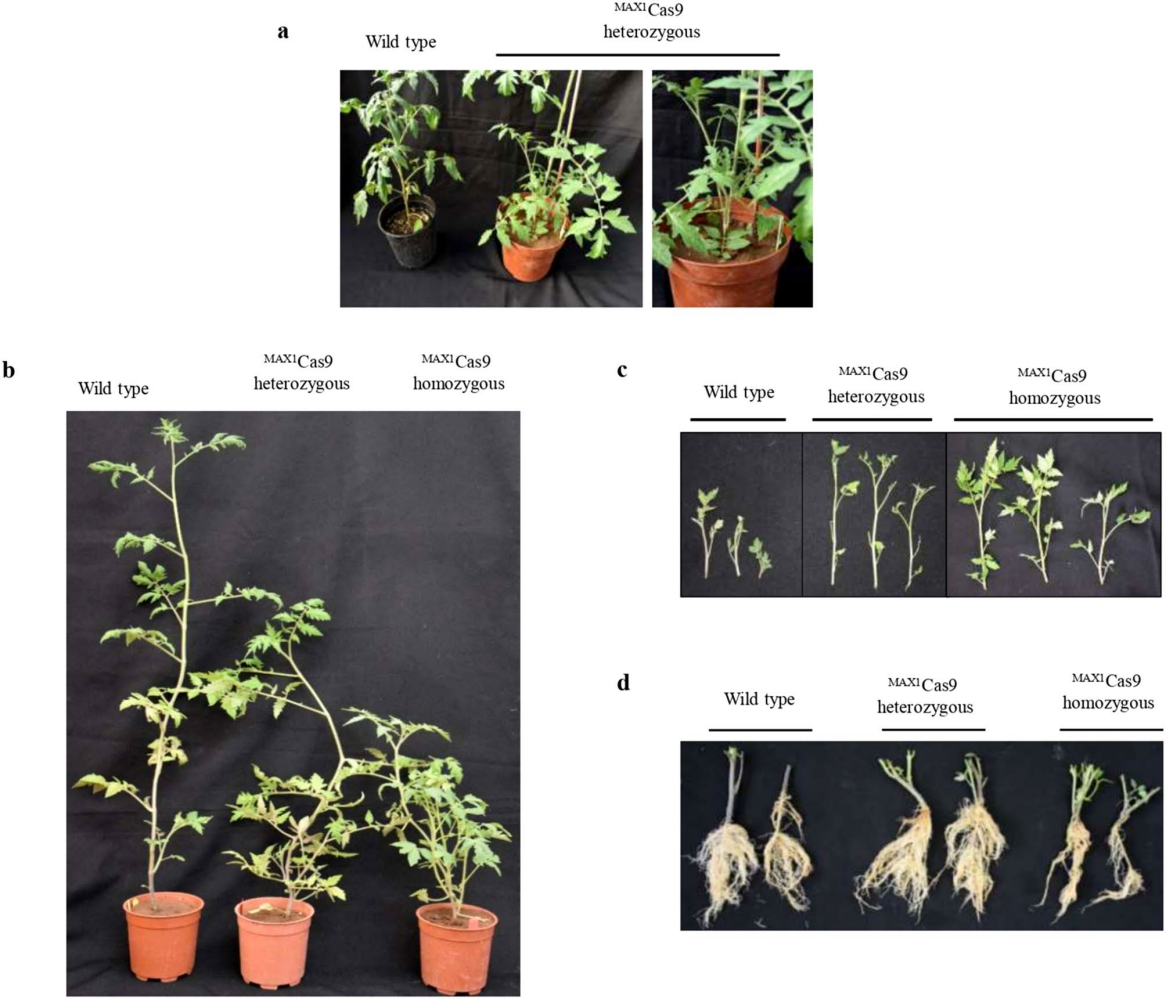
Morphological phenotype associated with *MAX1* edited tomato lines. (**a**) MAX1-Cas9 edited T_0_ heterozygous plant showing more growth of axillary buds as compare to wild type. (**b**) T_1_ heterozygous plant showing mild change in phenotype. Wild type plants with normal vegetative growth; MAX1-Cas9 edited T_1_ heterozygous and homozygous T_1_ line (**c, d**) Comparison of length of axillary branches & root morphology in the wild type and Cas9 mutants.

**Figure 4:**
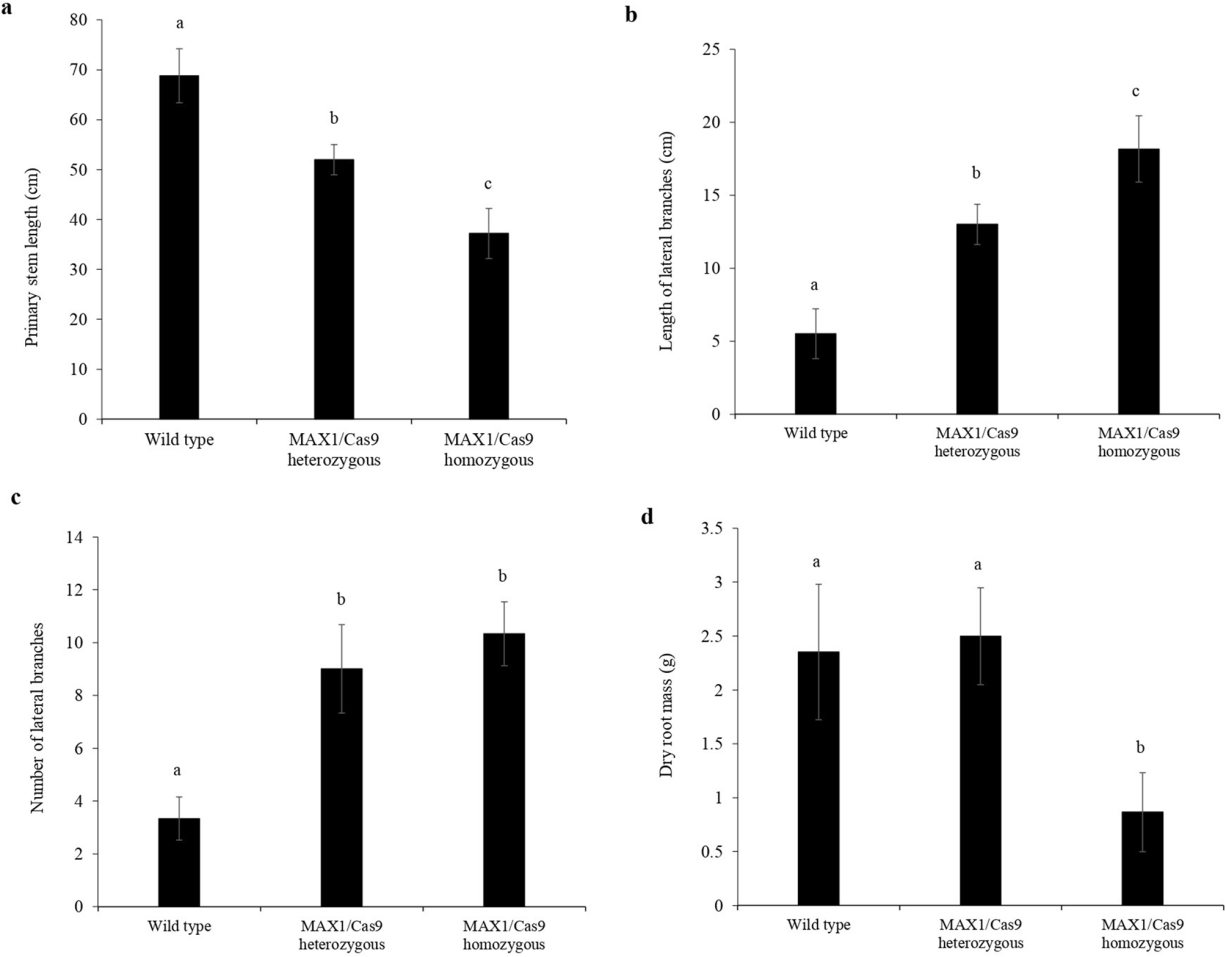
Quantitative analysis of morphological phenotypes in *MAX1* edited lines. (**a**) Quantitative estimate of primary stem length in 1-month-old MAX1-Cas9 mutated tomato plant average ± SE. (**b**) Length of lateral branches after 1 month of growth, average ± SE. (**c**): Number of lateral branches after 1 month of growth, average ± SE. (**d**) Quantitative estimate of dry root mass of wild type and MAX1-Cas9 mutant plants after 2 months of growth. Values are the average ± SE (n = 6). (Level not connected by same letters are significant, p < 0.05; Student’s t test).

Previous studies with RNA silencing of *MAX1* in tomato suggest that *max1* mutants exhibit an increase in shoot branching, lateral roots and overall dwarfing^29^. Surprisingly, in our study, the heterozygous lines had shown more growth of axillary buds as compare to control even in T_0_ generation (Fig. 3a). Consistently, we also observed similar phenotypic changes in the MAX1-Cas9 mutated homozygous T_1_ plants such as decreased plant height (Figs. 3b, 4a), branched shoots and more growth of axillary buds (Figs. 3c, 4b), which are significantly increased in number as compared to the wild type plants (Figs. 4c, S2), additionally the mutated plants have a significant difference in their dry root mass as compared to control plants (Figs. 3d, 4d). In conclusion, we observed characteristic feature of SL biosynthesis defective mutant in the MAX1-Cas9 heterozygous T_1_ plants as compared to wild type plants but these phenotypic changes were mild in nature with respect to homozygous plants.

### *MAX1* mutated host plant reduces germination of root parasitic weed *P. aegyptiaca*

**Figure 5:**
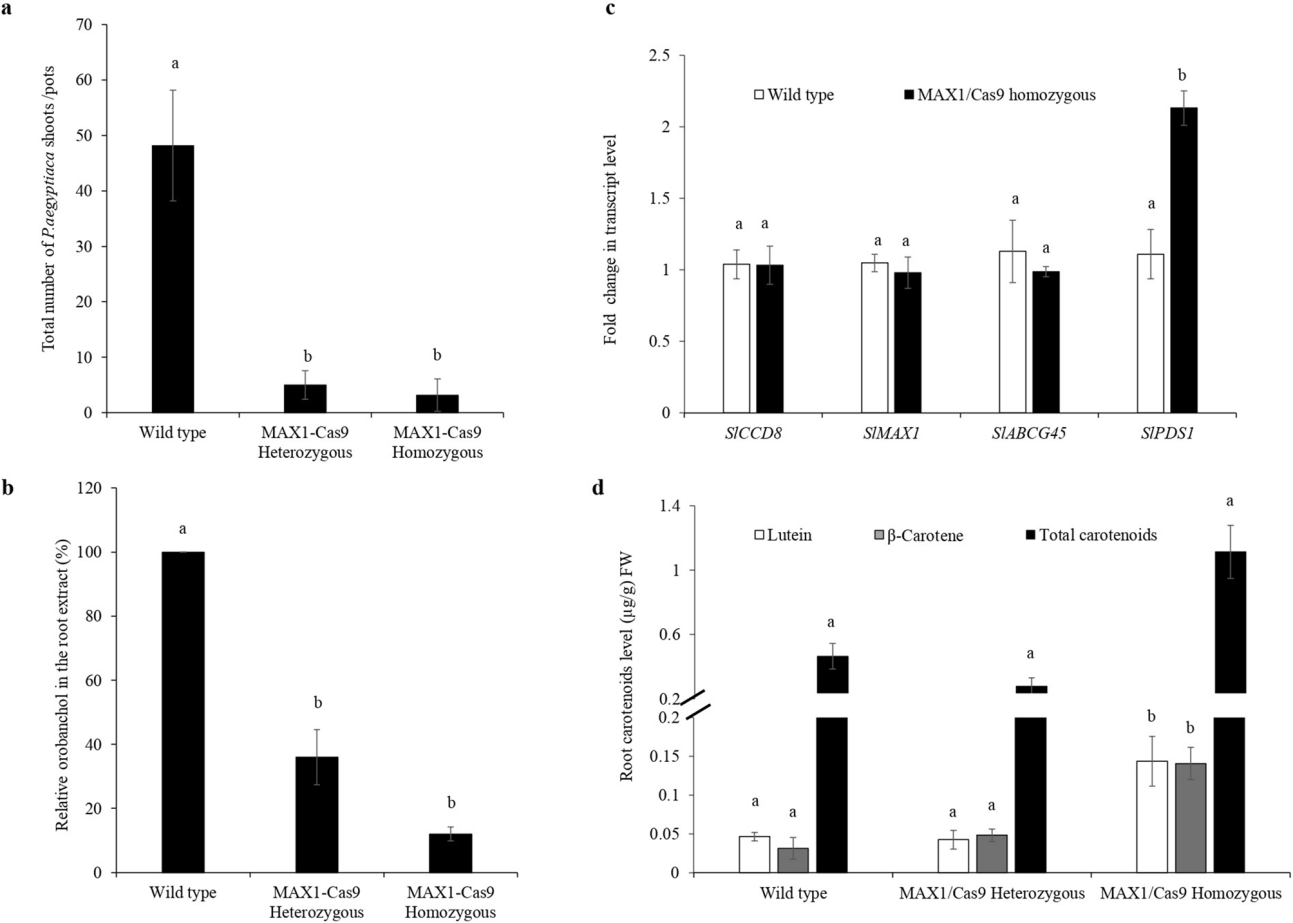
Resistance to parasitic infestation, real time PCR and carotenoid content analysis. To evaluate resistance to the root parasitic weeds, host roots of the tomato wild-type and MAX1-Cas9 mutated T_1_ lines were rinsed after 3 months of infestation with *P. aegyptiaca* seeds. Tubercles larger than 2 mm in diameter were counted for analysis. (**a**) Average number of *P. aegyptiaca* tubercles and shoots attached to the MAX1-Cas9 mutants and wild type tomato plants in the pot assay. Bars represent average of two experiments with six independent plants of each T_1_ mutants ± SD values. (**b**) Orobanchol contents in the roots of tomato MAX1-Cas9 edited plants as compared to wild type. LC–MS/MS analysis was done three times with three biological samples from each mutant. Data represented as average ± SD n (3). (**c**) Real time PCR analysis of *CCD8, MAX1, ABCG45* and *PDS1* transcript levels in the root of MAX1-Cas9 edited homozygous lines and wild type plants. Fold change in transcript level was shown after normalization with internal control tomato elongation factor-1α (EF1-α). Bars with different letters are significantly different from each other (student t-test at p < 0.05 when compared with the wild type). Result shown represent the mean of three experimental repeat ± SE. (**d**) Quantitative estimate of carotenoids content in the tomato root of wild type and MAX1-Cas9 mutant plants. Values are based on the analysis of 2 months old plants grown in green house with optimum conditions. Carotenoid analysis was done three times with three biological samples from each mutant. Data represented as average ± SD n (3). Statistical differences were calculated with Student’s one-tailed t-test (p < 0.05). Different small letter on the bar indicate a significant difference between the MAX1-Cas9 edited lines as compared to the wild type plants.

To evaluate, whether the MAX1-Cas9 mutated line show reduced germination of *P. aegyptiaca*, we selected MAX1-Cas9 heterozygous and homozygous tomato lines from T_1_ generation, and mixed with *P. aegyptiaca* seeds (15 mg/kg soil) and grown for three months in a greenhouse with optimum control conditions. To analyze the resistance, we counted only fresh and viable parasite tubercles and shoots that are larger than 2 mm in diameter. Results obtained suggested that the total number of germinated parasite tubercles and shoots were significantly reduced in the MAX1-Cas9 mutated heterozygous and homozygous plants as compared to the wild-type plants (Fig. 5a). To further correlate resistance to *P. aegyptiaca* infection and SL content of the MAX1-Cas9 mutated plants, we analyzed the total orobanchol content in the root extract of wild-type and MAX1-Cas9 edited host plants. We observed a significant reduction of total orobanchol content in both heterozygous and homozygous plants as compared to the wild type. However, the decrease in orobanchol was more pronounced in homozygous as compared to heterozygous mutant plants (Fig. 5b).

### MAX1-Cas9 mutation affects upstream carotenoid-biosynthesis pathway

SLs are carotenoid derivatives and carotenoids are isoprenoid pigments that differ in structure and stereometric configuration with a system of conjugated double bonds that is responsible for their colors^9,30^. In plants, SL and carotenoid biosynthesis take place mainly in the plastids^31^. It is initiated by the condensation of two molecules of geranylgeranyl diphosphate and with a series of reactions in presence of different enzymes, they produce diverse active molecules such as lycopene, lutein, *β* -carotene and violaxanthin^9,32^**.** To assess whether targeted mutagenesis of *MAX1* gene induces feedback regulation and affect transcript level of SL biosynthetic gene *CCD8* and *MAX1,* expression analysis was performed in homozygous lines using quantitative real-time PCR, however, we did not observe any significant difference in the expression of *CCD8* and *MAX1* transcript (Fig. 5c)*.* This data suggests that mutagenesis of *MAX1* does not affect expression of *CCD8* or the feedback pathway*.* Previous studies with *Petunia hybrida* ATP Binding Cassette transporter explored that *PDR1* act as SL transporter which has a key role in regulating the growth of axillary branches and symbiotic interactions with arbuscular mycorrhizae. Additionally, *P. hybrida pdr1* mutants are defective in SL exudation from their roots, resulting in reduced germination of *P. ramosa*^33^. To investigate whether the resistance against root parasitic weed depends on SL exporter the expression of *ABCG45,* a homolog of *PDR1* in tomato, was analyzed in *MAX1* mutants and we did not observe any significant change in the transcript, which suggest that *ABCG45* is not involved in parasitic weed resistance mechanism (Fig. 5c). Further, to explore whether defective SL biosynthesis in MAX1-Cas9 mutant affects the behavior of the upstream carotenoid-biosynthesis pathway, we analyzed the expression of phytoene desaturase-1 (*PDS1*), a gene involved in the biosynthesis of *β*-carotene^30,34^. Our results demonstrated that the expression of *PDS1* is significantly upregulated in MAX1-Cas9-edited homozygous T_1_ lines as compared to the wild type (Fig. 5c). To further validate the *PDS1* expression data, we analyzed the total carotenoids content in the root of *MAX1* mutated plants. Our results suggest that homozygous mutants have substantially increased content of total carotenoids along with *β*-carotene and lutein as compare to heterozygous and wild type plants (Fig. 5d).

## Discussion

Plant-parasitic weeds exert biotic stresses and create significant constraints for farming and food production worldwide. Root parasite *Phelipanche* and *Orobanche* spp. attacks many economically important crops throughout the Mediterranean and semi-arid regions and are regarded as the most serious pests^35^. Hence, to control parasitic weeds effectively, an innovative solution is urgently needed. A major goal of plant genome editing is to improve crop yield and quality. Studies on host plant–parasitic weed interactions have explored many key host genes associated with parasitic weed resistance^36–39^.

In this study, using CRISPR/Cas9 genome-editing system, we have developed a non-transgenic *MAX1* mutant of tomato that exhibits reduced orobanchol content and increased resistance to *P. aegyptiaca*. We designed a sgRNA to target the third exon of the tomato *MAX1* gene to disrupt SL biosynthesis. In Agrobacterium transformed T_0_ transgenic line, we found editing events in the *MAX1* targeted locus in one (Line 1) out of five lines (Fig. 1). The heritability of the mutation and the generation of transgene-free plants are major concern, when using the CRISPR/Cas9 system^40,41^. To avoid the somatic nature of editing events, T_0_ line 1 was self-pollinated to generate T_1_ transgenic plants. In the T_1_ generation, we observed that the mutations induced in T_0_ line 1 are stably inherited by the T_1_ generation, without any new mutations (Fig. 2).

Using sanger DNA sequencing of potential off-target sites with mismatches of fewer than 4nt with MAX1-sgRNA, we did not identify any off-target mutation (Fig. S1 and Table S2). The specificity of Cas9 is greatly influenced by various factors and non-specific off-target site cleavage is a major challenge in the use of the CRISPR system^42^. In higher plants, undesirable mutations resulting from the CRISPR/Cas9 system are generally rare, however, non-specific undesired mutations can be avoided using more specific sgRNAs^43–47^.

SLs play a major role in controlling plant architecture, regulating shoot branching and to influence lateral and adventitious root formation^48^. Recent studies on the morphology of tomato *max1*-mutant plants by gene silencing have shown an increase in shoot branching, reduced plant height and increased adventitious roots formation^49^. Interestingly, in our study MAX1-Cas9 heterozygous tomato plants shown intermediate phenotype with respect to homozygous and wild type plants. These heterozygous plants displayed reduced plant height, increased number of axillary branches, nodes and adventitious roots compared to the wild-type plants (Figs. 3, 4). However, morphological phenotypic changes associated with heterozygous lines were mild in nature than homozygous plants.

In the view of above, an argument can be raised that transgenic plants bearing Cas9 expression cassette in their genome constitutively express Cas9-sgRNA throughout their life and mutation could be accumulated during growth that will affect phenotype in heterozygous plants. To support our results, we showed that homozygous plants generated from the same line showed no off-target effects. Moreover, our *MAX1* heterozygous plant shows a characteristic phenotype of SL defective mutant though it is mild in nature. These results strongly support our data and provides an explanation of phenotypic defect in *MAX1* heterozygous. Another explanation for this could be the heterozygous mutants (in-frame 9nt deletion) due to the absence of amino acid Lys252-Arg253-Iso254 in the mutated protein behaving as semi-dominant negative or it could be a dosage gene effect, where one normal copy of gene exists and one copy mutated, that could affect the normal growth of plants if the protein has a role in important cellular function however these explanations need to be thoroughly investigated.

To evaluate host resistance to *P. aegyptiaca*, we used a pot system containing soil infested with *P. aegyptiaca* seeds as described previously^50^. In the current study, coincidently with the suppression of SL content, a significant decrease in the number of total germinated parasite tubercles and shoots was observed in *MAX1* mutated plants as relative to the wild type plants. In contrast, the wild-type plants were highly susceptible to the parasite infestation (Fig. 5a). Since orobanchol is a major SL in tomato root exudates^51^, and acts as a specific germination inducer for *P. aegyptiaca,* hence we determine the orobanchol content in the root extract of the MAX1-Cas9 mutated lines. Orobanchol content was found to be significantly reduced in the MAX1-mutated lines relative to the wild type (Fig. 5b).

Previously it has been reported that SLs are able to modulate local auxin levels, and the action of SL is dependent on the auxin status of the plant, suggesting that specific SL content influences the expression of genes involved in auxin biosynthesis^52^. Another study reported that an increase in transcript level of SL biosynthetic gene *D27* and *CCD8* in tomato roots occurs after parasitic weed infection, suggesting SL-biosynthesis pathway gets activated after parasitic infection^53^. Several studies reported that SL-biosynthetic pathways are strictly regulated by negative-feedback inhibition such as *D10* transcript levels are elevated in the dwarf mutants (*d10-1*) of rice, similarly transcript level of *RMS1* are enhanced in *ramosus* mutants of pea and application of synthetic SL GR24 restored the transcripts level^54–56^. To evaluate the role of *MAX1* in feedback regulation and mechanism of resistance, we analyzed the transcript level of *CCD8*, *MAX1* and *ABCG45* (Fig. 5c). However, no alteration in transcript level was observed suggesting that possibly *MAX1* did not involve in the control of feedback inhibition of SL pathway or carlactone accumulation has no role in modulation of SL biosynthetic pathway. Moreover, the expression of SL exporter *ABCG45* was also unaffected. Interestingly in our study *MAX1*-mutated homozygous lines have increased levels of a carotenoid biosynthetic gene *PDS1* and total carotenoids (Fig. 5d). These results indicate that block in the SL biosynthesis pathway positively affects carotenoid biosynthesis possibly due to interconnection between SL production and carotenoid biosynthesis.

Previous studies from our laboratories demonstrated the movement of mobile exogenous siRNA from the host to the root parasites^50^, this provides an insight that, CRISPR/Cas9 genome editing technique could be used against the parasite itself by indirectly transforming parasite-specific sgRNA in the hosts, if the host gene does not share sufficient homology with the targeted sequences of the parasite. The unwanted cleavage due to the non-specificity of the sgRNA within the genome exerts significant limitations to the CRISPR/Cas9 system which can alter the function of a gene or induce genomic instability. Currently, several naturally occurring and genetically modified Cas9 enzymes and specific sgRNA designing tools have been developed to enhance site specific target cleavage^57–59^, however, the off-target effect is still considered as a major limiting factor^60^. In the current study, we demonstrate that genetic resistance to root parasitic weeds can be obtained using CRISPR/Cas9 mediated targeted mutagenesis of the *MAX1*, a SL biosynthetic gene in tomato. A similar strategy could be effectively used against other parasitic weeds to generate host resistance.

## Experimental procedures

### Materials and growth conditions

Tomato (*Solanum lycopersicum*) cultivar MP-1 was chosen for generation of transgenic plants. Tomato seeds were surface-sterilized using 1% bleach with tween-20 and 70% ethanol and grown on half strength Murashige and Skoog (MS) basal medium (CAISSON Laboratories, USA) containing 1.5% sucrose (Sigma), pH 5.8 and 7 g/L phytagel (Sigma). The *P. aegyptiaca* seeds were collected from infected field in Northern Israel and used to infest tomato host plants.

### sgRNA design and Cas9 vector construction

The tomato *MAX1* (*Solyc08g062950*) gene was chosen as the target and sgRNA sequence of 20 nucleotides was designed using the CRISPR-P webtool and cloned into the plant binary vector. The binary vector contains nptII gene as kanamycin selection marker, under the control of the NOS promoter. sgRNA sequence together with the guide scaffold, was amplified using forward primer containing *Sal*I site as part of the U6 Arabidopsis promoter and a reverse primer of the Pol III-terminator sequence that contained a *Hind*III site and pRCS35S:Cas9-AtU6:sgRNA-PDS was used as a template. The amplified DNAs (130 bp) were cloned into *Sal*I and *Hind*III digested pRCS-35S:Cas9-AtU6:sgRNA-PDS vector^61–63^. The verification of positive clones was done using diagnostic PCR and sequencing. The construct was transformed into tomato cultivar MP-1 using Agrobacterium tumefaciens strain EHA105.

### Agrobacterium-mediated transformation of tomato plants

The transformation of tomato was conducted as previously described, with slight modifications^64^. The cotyledons were pre-cultured for 2 day in dark with Murashige and Skoog (MS) medium containing 100 μM Acetosyringone, 0.1 mg/L IAA, 1 mg/L Zeatin and 0.7% phytagel subsequently infected with the Agrobacterium strain EHA105 (optical density at 600 nm less than 0.5) by immersion for 20 min. The explants were co-cultivated with Agrobacterium strain EHA105 for two days in dark and then transferred to the MS medium supplemented with 0.1 mg/L IAA, 1 mg/L Zeatin, 100 µg/ml Kanamycin, 300 µg/ml timentin and 0.7% phytagel for a week. Kanamycin-resistant shoots regenerated were transferred to tissue culture bottles containing the subculture medium with 0.1 mg/L Zeatin. Developed vegetarians were transferred to the half strength MS rooting substrate with the addition of 2 mg/L IBA. After three months, Kanamycin-resistant plantlets were obtained and used for subsequent analysis.

### Genotyping of transgenic plant

For genotyping, tomato plant leaves or roots genomic DNA was extracted using plant genomic DNA extraction kit and the genomic DNA flanks containing the sgRNA target sites were amplified using the specific primers SlMAX1-Int-F & SlMAX1-Int-R and then run on a 2% agarose gel using electrophoresis. Image was acquired using DNR Mini Lumi with UV light system. Direct sequencing of PCR products was done using appropriate primer. For PCR product cloning, pGEM-T kit from Promega were used.

### Analysis of off-target mutations

The potential off-target sites associated with sgRNA target sequence were analyzed with the CRISPR-P program^65^. Three off target sites with the highest probability score were selected. The genomic region flanks upstream and downstream to the off-target sites (300–400 bp) was amplified and sequenced with specific primers using sanger DNA sequencing (Table S3).

### Evaluation of *P. aegyptiaca* resistance assay

Resistance analysis of MAX1-Cas9 mutant tomato lines to the parasite was done as reported earlier ^66^. For infection one-month-old tomato seedlings were transferred into pots containing soil with a peat moss to perlite mixture ratio of 3:1, infested with seeds of *P. aegyptiaca* (15 mg/kg soil) and grown in a greenhouse under natural light with an average 14 h of daylight and a temperature of 20 ± 6 °C. Tomato plant roots from wild type and Cas9 mutant plants were collected, 3 months after exposure to the *P. aegyptiaca* seeds. The total number of viable *P. aegyptiaca* tubercles larger than 2 mm diameter attached to host roots were counted.

### RNA isolation and quantitative real-time PCR

Total RNA from tomato roots was extracted using spectrum plant total RNA kit (Sigma- STRN50-1KT) according to the manufacturer’s protocol. 500 ng of total RNA was used to obtained cDNA according to the protocol of Quanta Bioscience cDNA Synthesis Kit. Quantitative real-time PCR (qRT-PCR) was performed in a volume of 10 μl using PerfeCTa SYBR Green FastMix ROX (Quanta biosciences) with 5 times diluted cDNA as template. Tomato elongation factor 1-α was used as an internal control gene. Specificity of the primers was confirmed by melting curve analysis. The generated Ct values of target genes were normalized to the Ct value of internal reference EF1-α gene. Relative expression was calculated using 2^−ΔΔCt^ method and expressed as fold increase with respect to control^67^.

### SL extraction and analysis using HPLC–MS/MS

The SL extraction and quantification were performed as described previously^68^. Lyophilized tomato roots were grounded into fine powders using liquid nitrogen and extracted with the ethyl acetate extraction method. The tissues were transferred to a 4–10 times volume of ethyl acetate. The flask was treated with an ultra-sonic bath for a few minutes and then placed in a cool place (4 °C) for 2–3 days. The tissues are filtered off and washed well with ethyl acetate. The combined ethyl acetate was washed with 0.2 M K_2_HPO_4_ or saturated NaHCO_3_ to remove acidic compounds. The extract was dried over anhydrous MgSO_4_ or Na_2_SO_4_, filtered and the solvent was evaporated under reduced pressure. Samples were dissolved in 2 ml acetonitrile or methanol: water (25:75 v/v) and the quantification of orobanchol in extracts was performed using LC–MS/MS using orobanchol as standard.

### Carotenoid extract analysis

Carotenoid extract analysis was performed as reported earlier^69^. In brief, 1.5 g of fine grinded roots was used for extraction in presence of 8 ml hexane: acetone: ethanol (50:25:25 v/v), followed by 5 min of saponification in 1 ml of 8% (w/v) KOH. After addition of 1 ml of NaCl (25%), the saponified material was extracted twice with hexane, which was then evaporated in speed vacuum. The solid pellet was resuspended in 400 μl of ACN: MeOH: DCM (45:5:50 v/v) and passed through a 0.2 μm Nylon filter before HPLC analyses. Two independent biological samples from each line were pooled for carotenoid analysis.

### Statistical analysis

For statistical analysis, experiments were performed independently at least three times using three biological repeats and the results are expressed as mean ± SD. Statistical significance difference was analyzed by Student’s t-test using JMP Pro 14 software. A p-value of < 0.05 was used as a cutoff for statistical significance that is indicated with different letters above the bar.

## Supplementary Information

Supplementary Information
