# Supplementary material for: CRISPR/Cas9 mediated mutagenesis of MORE AXILLARY GROWTH 1 in tomato confers resistance to root parasitic weed Phelipanche aegyptiaca
Source: Sci Rep. 2021 Feb 16;11:3905. doi: 10.1038/s41598-021-82897-8 (PMC7887253; doi:10.1038/s41598-021-82897-8)
Supplement: Supplementary file 1 — Supplementary Information [file 41598_2021_82897_MOESM1_ESM.pdf]

Supplementary Information:

CRISPR/Cas9 mediated mutagenesis of *MORE AXILLARY GROWTH 1* in tomato confers resistance to root parasitic weed *Phelipanche aegyptiaca*

Vinay Kumar Bari[1, 2*], Jackline Abu Nassar[1], Radi Aly[1*]

[1]Department of Plant Pathology and Weed Research, Newe Ya'ar Research Center, Agricultural Research Organization (ARO), Ramat Yishay, Israel.

[2]Current Address: Department of Biochemistry, School of Basic Sciences, Central University of Punjab, VPO-Ghudda, Bathinda, India.

*Correspondence

Vinay Kumar Bari: vinay.bari@cup.edu.in

Radi Aly: radi@volcani.agri.gov.il

MAX1-sgRNA target CAAGTTCTCAAAAGAATCCCGGG

Off target (4 mismatch) AAAATTTTCAAAAGAATACCTGG

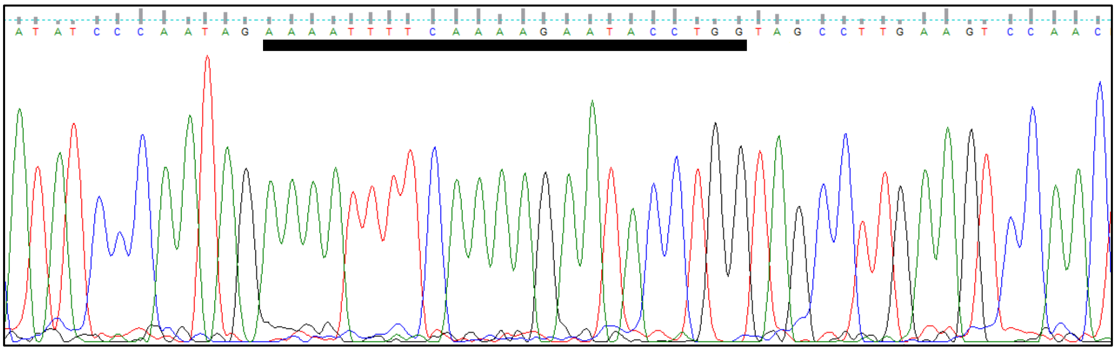

Fig. S1. PCR product DNA sequencing chromatogram of potential off-target of the MAX1-sgRNA. No mutation at off-target site detected in the genome of MAX1-Cas9 edited homozygous lines. Region highlighted in black bar correspond to off target sites.

Wild type MAX1-Cas9 Heterozygous MAX1-Cas9 Homozygous

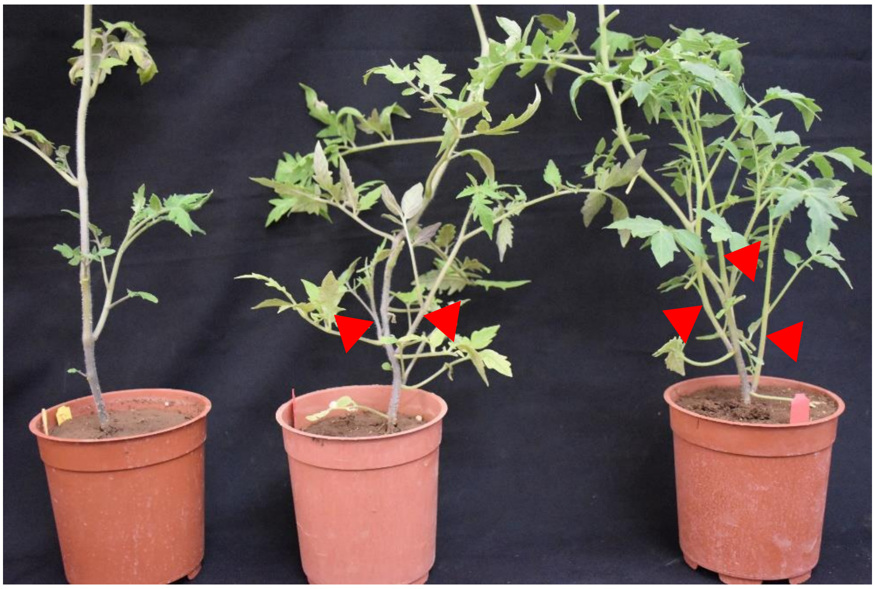

Fig. S2. Characteristic axillary bud outgrowth phenotype of 1-month-old MAX1-Cas9 heterozygous and homozygous T_1 plants as compare to wild type tomato plants grown in green house.

Table S1. Segregation pattern of MAX1-Cas9 mutation from T_0 to the T_1 generation. The zygosity of heterozygote in T_0 plant lines was putative. +, Foreign DNA (nptII) was detected; -, Foreign DNA (nptII) was not detected.

MAX1-Cas9 Line	T_0 generation		T_1 generation		
	Mutation type	Zygosity	Mutation type/ total test	Mutation exists no./ total test (%)	Foreign DNA (nptII) segregation (%)
Line 1	-9nt	Heterozygous	homozygous (5/24), heterozygous (12/24), wt (7/24)	17/24 (70.8%)	9+; 5- (35%)

Table S2. Mutational analysis of off-target sites predicted to associated with MAX1-sgRNA edited lines. To analyse the off target using CRISPR-P, high score value was considered as top hit. The PAM motif (NGG) is marked by blue; mismatching bases are shown in red colour.

Name of putative off -target sites & score	Putative off-target locus	Sequence of the Putative off-target site	Number of mismatching bases	Presence of mutation detected
OFF1 (0.394)	SL2.50ch09:+61329926 Solyc09g064200.2 (intron)	AAAATTTTCAAAAGAATACC**TGG**	4	0
OFF2 (0.331)	SL2.50ch01:+47873454 Intergenic	AAAGTTCTCTAAAAAATCCA**TGG**	4	0
OFF3 (0.321)	SL2.50ch08:-36699521 Intergenic	AAAGTTATCAAAAGAATCAC**AGG**	3	0
OFF4 (0.318)	SL2.50ch04:-54647171 Intergenic	AAAATTGTCAAAAAAATCCC**TGG**	4	0

Table S3. List of primers used in this study

Oligo's name	Sequence (5' ⟶ 3')	Use
U6R-HindIII	TAAGCTAAGCTTCGATCTAAAAAAAGCACCGACT	MAX1 Exon-3 targeting sgRNA cloning in Cas9 expression vector
MAX1sgRNA-F	AGAGTCGACATAGCGATTCAAGTTCTCAAAAGAATCCCGTTTTAGAGCTAGAAATAGCAAG	
RCSMAX1-DG-F	CAAGTTCTCAAAAGAATCCC	Diagnostic PCR and sequencing
sgRNA-SEQ-F	GGACACTGACGGCTTTATG	
sgRNA-SEQ-R	GACGAACGGATAAACCTTTTC	
Npt-II-F	TCTTGTCGATCAGGATGATC	Transgene validation
Npt-II-R	AGAAGAACTCGTCAAGAAG	
pcoCas9-F	GATTGGCTTGATGAACTTGTAG	
pcoCas9-R	ATGATGATCTTGATAACCTTCTTG	
TmMAX1-Int-F	GAGGGAGATCTCACATTCTCTG	Mutation detection and sequencing
TmMAX1-Int-R	CCAGCAAGTGCTCATAAGTTAC	
TmMAX1-Nest-F	GGTGTGGATTTTGGACTTTC	
SlCCD8-qF	CCAATTGCCTGTAATAGTTCC	Real time PCR
SlCCD8-qR	GCCTTCAACGACGAGTTCTC	
ABCG45-qF	TGATTATCCAAGACCAGAGCTG	
ABCG45-qR	GCACCAGCAATAACAATCTCC	
SlPDS1-qF	TGATTATCCAAGACCAGAGCTG	
SlPDS1-qR	GCACCAGCAATAACAATCTCC	
SlEFα1-qF	GATTGGTGGTATTGGAACTGTC	
SlEFα1-qR	AGCTTCGTGGTGCATCTC	
SlMAX1-qF	GCAAGGGAGTCAGAGAAGTTAG	
SlMAX1-qR	GATGGCAAGCAACCAGATAGA	
OFF9-F	CTCCTGAAGTTTTAGATGGAAG	Off target PCR and sequencing analysis
OFF9-R	AGGCTATGTTCAGAAAGCTAAC	
OFF1-F	TATTTAAGGTAAGGTAAG	
OFF1-R	ATTCACCATAACTGAG	
